# Neurotrophin3 promotes hepatocellular carcinoma apoptosis through the JNK and P38 MAPK pathways

**DOI:** 10.7150/ijbs.72982

**Published:** 2022-10-03

**Authors:** Zhangshuo Yang, Hao Zhang, Maohui Yin, Zhixiang Cheng, Ping Jiang, Maohui Feng, Bo Liao, Zhisu Liu

**Affiliations:** 1 Department of Hepatobiliary and Pancreatic Surgery, Zhongnan Hospital of Wuhan University, Wuhan 430071, Hubei, People's Republic of China.; 2 Department of Gastrointestinal Surgery, Zhongnan Hospital of Wuhan University, Wuhan 430071, Hubei, People's Republic of China.

**Keywords:** Hepatocellular carcinoma, Neurotrophin 3, p75NTR, Tumor suppress

## Abstract

Although liver cancer is a malignant tumor with the highest mortality across the world, its pathogenesis and therapeutic targets remain unclear. Apoptosis, a natural cell death mechanism, is an important target of anticancer therapy. The discovery of effective apoptotic regulators can lead to the identification of novel therapeutic targets for treating cancer. Neurotrophin 3 (NTF3) is a member of the nerve growth factor (NGF) family that is involved in the progression of various cancers, including medulloblastoma, primitive neuroectodermal brain tumors, and breast cancer. NTF3 is under-expressed in human hepatocellular carcinoma (HCC), albeit its specific effects and the action mechanism have not been elucidated. Here, we confirmed that NTF3 expression was significantly low in HCC with reference to the GSEA database. By collecting patient data from our center and performing qRT-PCR analysis, we found that *NTF3* expression was significantly downregulated in 74 patients with HCC. Low NTF3 expression was associated with a shorter overall survival (OS), recurrence-free survival (RFS), progression-free survival (PFS), and disease-specific survival (DSS). Both *in vivo* and *in vitro* experiments revealed that NTF3 considerably inhibited the progression of HCC cells. We found that the ligand NTF3 is regulated by c-Jun and binds to the p75 neurotrophin receptor (p75NTR) and then activates the JNK and P38 MAPK pathways to induce apoptosis. Entinostat (the target of HDAC1/HDAC3) can activate the NTF3/p75NTR pathway. These results indicate that NTF3 is a tumor suppressor, and that its low expression can help in predict poor clinical outcomes in HCC. Therefore, NTF3 can be used as a potential treatment molecule for HCC.

## Introduction

Cancer is a major public health problem globally. Liver cancer is predicted to be the sixth most frequently diagnosed cancer and the fourth-leading cause of cancer death worldwide in 2021 1,2. In China, the recurrence rate of liver cancer is high, and the effectiveness of the conventional treatment is poor 3. Hepatocellular carcinoma (HCC) is the most common type of liver cancer (90%), and the remaining types include cholangiocarcinoma and mixed-type tumors 4. The pathogenesis of HCC is complex, involving the regulation of several oncogenes and tumor suppressor genes, as well as abnormal cell signals. It is therefore necessary to continue to study its molecular mechanism to better improve the diagnosis and treatment of HCC.

Apoptosis is also known as programmed cell death and it helps maintain a balance between cellular proliferation and death 5. Cells undergoing apoptosis isolate themselves from the neighboring, healthy cells in a controlled manner, thereby minimizing damage and destruction to the neighboring cells 6,7. Apoptosis is considered a good option for cancer treatment 8. The discovery of molecules that effectively stimulate apoptotic pathways can help identify better therapeutic targets for the treatment of HCC.

Neurotrophins (NTs) are a type of growth factor that controls the growth and differentiation of tumor cells. Nerve growth factor (NGF) is composed of a variety of proteins, including the prototype member NGF; human ciliary neurotrophic factor (CNTF); brain-derived neurotrophic factor (BDNF); NTF3, NT4/5, and NT-7 (reported only in invertebrates), glial-derived neurotrophic factor (GDNF); and neuroturin (NRTN). They bind to the two types of receptors: tropomyosin tyrosine kinase receptor (Trk) and the common NT receptor p75NTR 9,10. As a new tumor suppressor, NTF3 is a member of the neurotrophic family of ligands. It can bind to p75NTR to activate the signaling pathway linked to apoptosis. Past studies have shown that p75NTR-dependent apoptosis is related to Rac and Jun kinase (JNK) activities and caspase activation 11. The NTF3/TrkC signal inhibits growth in medulloblastoma (MBL) 12,13. In primitive neuroectodermal brain tumors (PNETS), TrkC accelerates apoptosis and terminal neuronal differentiation via its ligand NTF3 14,15. NTF3 can promote the growth of brain metastatic cells from breast cancer via HER2 16. The low expression of NTF3 in HCC was previously reported to be associated with poor prognosis 17, although the inhibiting effect of NTF3 on HCC and its specific action mechanism has not been fully elucidated.

In this study, we found that NTF3 is a tumor suppressor protein in HCC that is regulated by c-Jun and histone deacetylation. It binds to p75NTR and promotes apoptosis through the JNK and P38 MAPK pathways toward inhibiting tumorigenesis and development.

## Materials and methods

### Clinical specimens

All samples were sourced from the sample collection library of the Wuhan University Zhongnan Hospital and received the ethical approval of the institute. All participants provided their signed written informed consent. In this study, all patients did not receive preoperative radiotherapy or chemotherapy. Two pathologists who were not involved in this study confirmed the HCC organization.At the time of sample collection, the RNA later protector was used for the subsequent qRT-PCR test, and the samples were stored in liquid nitrogen for subsequent Western blotting analysis.

### Cell culture, lentiviral construction, and cell transfections

The cells used in this study were purchased from the Shanghai Chinese Academy of Sciences Cell Bank and cultured in high-glucose DMEM (Gibco,CA,USA) supplemented with 10% fetal bovine serum (TICO,CA,USA) and 1% penicillin-streptomycin reagent (Gibco,CA,USA), and incubated under 5% CO_2_ atmosphere at a constant temperature of 37°C. A lentivirus was next constructed using cells at a density of 30% and HiTransA reagent (Genechem, Shanghai, China) to transfect Huh-7 with an MOI = 5 and HCCLM3 with an MOI = 10. Small interference RNA was synthesized by China JTS Scientific Company. Supplementary Material Table S1 provides siRNA and virus sequences.

### qRT-PCR

Trizol was used to extract total RNA from the cells. cDNA was synthesized through reverse transcription (Vazyme, Shanghai, China) using the BIO-RAD instrument and with SYBR Mix (Vazyme, Shanghai, China) to perform qRT-PCR.The information on the sequence is provided in Supplementary Material Table S2.

### Western blotting

The powerful RIPA (Biyuntian, Shanghai, China) cell lysate was used to extract the cell protein. The protein was then electrophoresed through 10% SDS-PAGE gel. After blocking with 5% skimmed milk for 1 h, it was incubated with the primary antibody overnight at 4°C followed by incubating with the corresponding secondary antibody for 1 h at room temperature. The ECL luminescent solution was used for development, and the Kodak medical X-ray film was used for scanning and archiving. Supplementary Material Table S3 provides the antibodies.

### Cell proliferation assays

In a 96-well plate, Huh-7 cells (3 × 10^3^/well) and HCCLM3 (2.5 × 10^3^/well) were added to 200 µL of the complete medium and 10 µL of the CCK8 reagent (Biosharp, Hefei, China). The logarithmic growth curve of the cells was inoculated in a 96-well plate as per the operating instructions of the EDU Kit (Ribobio, Guangzhou, China).

### Wound healing assay

A 6-well plate was inoculated with 1 × 10^6^ cells/well, cultured in a serum-free medium for 24 h, and then scratched with a 100-µL pipette tip. The images were then captured and observed at 0, 24, 36, and 48 h, respectively.

### Transwell assay

Using a 96-well plate, 2.5×10^4^ logarithmic growth phase Huh-7 cells and HCCLM3 cells were inoculated in the upper chamber (Corning, NYC, USA), after incubating for 2 days.

### Cell apoptosis analysis and cell cycle analysis

The cells (1×10^5^) cells were collected according to the manufacturer instructions of the Annexin V-FITC/PI or Annexin V-PE/7-AAD (AO2001-09A-G, Tianjin, China).

Then, 1×10^6^ cells were collected and processed according to the manufacturer instructions of the Cell Cycle Staining Kit (70-CCS012; MultiSciences, Hangzhou, China).

### In-vivo experiments

Five-week-old male BALB/c nude mice (provided by Charles River Company, China) were selected and raised with the SPF level in the Animal Experiment Center. The HCCLM3 cells were transfected with NTF3 (5×10^6^ cells) and Huh-7 cells with NTF3 knockdown (6×10^6^ cells) and then injected subcutaneously into the axillary position. The HCCLM3 cells (3×10^5^ cells) overexpressing NTF3 were injected into the tail vein. The lung tissues of the nude mice were collected and observed after 6 weeks.

### Endogenous Co-Immunoprecipitation (Co-IP)

The Huh-7 and HCCLM3 cells were cultured in a 10-cm^2^ petri dish. After extracting the protein with 0.1% TritonX100,sodium protosate,sodium fluoride and PMSF (Beyotime, Shanghai, China), 1/10 of the extract was used as the input solution, and the remaining extract was used as IP. To the cell lysate, 5 μL of p75NTR antibody was added and subjected to precipitation by incubation at 4 °C for 1 h. Then, 20 μL of magnetic beads were added, the reaction mixture was mixed well, and then incubated overnight at 4 °C. An appropriate volume of the washing buffer was then added to the centrifuge tube, the tube was placed on a vertical shaker, and washed for 3 min; after which the centrifuge tube was placed on a magnetic stand for 5-10 s, and the supernatant was discarded. After the final rinse, we prudently discarded the supernatant and added an appropriate volume of 2× SDS mercaptoethanol-containing loading buffer, followed by boiling for 10 min before loading the sample for the Western blotting.

### Immunohistochemistry

The paraffin sections of 4-mm thickness were prepared on glass slides, dehydrated with a gradient series of alcohol and xylene, and stained with the UltraSensitiveTM S-P Kit (Maixin, Fuzhou, China), as suggested by the manufacturer.

### Luciferase Reporter Assay

Construct pGL3-NTF3-WT, pGL3-NTF3-MUT plasmid was constructed and used to transfect the plasmid into MHCC97H and Huh-7. The cell lysate was collected and the dual luciferase reporter gene detection kit was used (RG027, Beyotime).

### Chromatin immunoprecipitation (ChIP)

To a total of about 1×10^7^ MHCC97H and Huh-7 cells, a final concentration of 1% formaldehyde (47608, Sigma, NJ, USA) and 0.125M glycine (62011516, Shanghai, China) were used for cross-linking and fixation. After collecting the cells, 720uL of the ChIP Lysis Buffe was added and the cells were lyse on an ice bath for 20 min.The supernatant was subjected to ultrasound and save as the input group,followed by division into the IgG group and IP groups. After immunoprecipitation, elution was performed and a DNA purification kit (CW2301, CWbiotech, Beijing, China) was used for DNA purification and recovery.

### Bioinformatics analysis

To develop heat maps and prognostic analysis, the liver cancer transcriptome and clinical data were obtained from TCGA, GEO, GEPIA (http://gepia.cancer-pku.cn), and LIHC databases. The survival curve was obtained from Kaplan-Meier Plotter (http://kmplot.com/analysis).

### Statistical analysis

The Graphpad prism8.0 and SPSS21.0 were used for statistical analyses. Parameter analysis was performed by Chi-square test、T-test and One-way ANOVA. P-value is assigned as *P < 0.05; **P < 0.01; ***P < 0.001.

## Results

### NTF3 is significantly downregulated in HCC and is also related to poor prognosis

**Figure 1:**
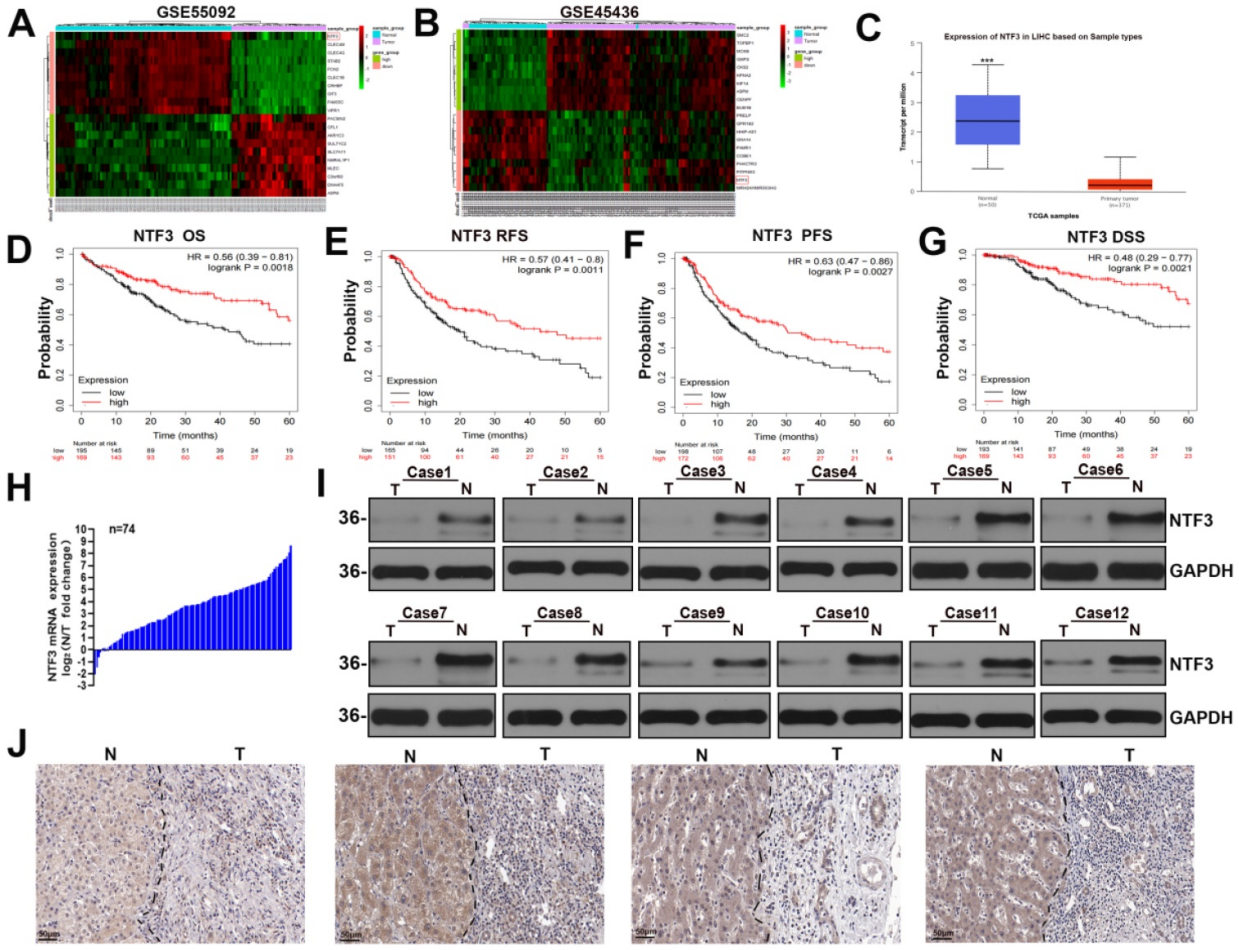
** NTF3 was significantly downregulated in HCC and was associated with a poor prognosis. A, B** The GSE45436 and GSE55092 datasets in the GEO database implied that the expression of NTF3 in the paracancerous tissues was significantly higher than that in the liver cancer tissues. **C** The expression of NTF3 in the liver cancer tissues (n = 371) and the adjacent tissues (n = 50) in the TCGA database. **D, E, F, G** Kaplan-Meier analysis suggested that the low NTF3 expression was associated with poor prognosis. **H** The qRT-PCR results of 74 patients proved that the level of *NTF3* mRNA in the adjacent tissues was higher than that in the cancerous tissues. **I** The level of NTF3 protein was observed in 12 sets of human HCC cancers tissues (T) and peritumor tissues (N). **J** NTF3 immunohistochemical (IHC) staining was performed for clinically identified HCC tumor specimens (T) and the adjacent non-tumor specimens (N). *P < 0.05; **P < 0.01; ***P < 0.001.

By analyzing the GSE45436 and GSE55092 datasets in the GEO and TCGA databases respectively, we found that NTF3 expression in the adjacent cancer tissues was significantly higher than that in the liver cancer tissues (Fig. 1A-C). The KM website and our center clinical data have proved that the low NTF3 expression was associated with poor prognosis (Fig. 1D-G) and is significantly correlated with the tumor size, staging, and grading (Supplement Table S4). Furthermore, the qRT-PCR results of 74 patients proved that the *NTF3* level in the adjacent tissues was higher than that in the cancerous tissues (Fig. 1H). WB and immunohistochemical analyses verified that the NTF3 protein levels in the adjacent tissues were considerably higher than those in the cancerous tissues (Fig. 1I, J).

### NTF3 overexpression suppresses the proliferation of HCC cells, reduces their migration and invasion ability, increases apoptosis, and induces cycle arrest *in vitro*

**Figure 2:**
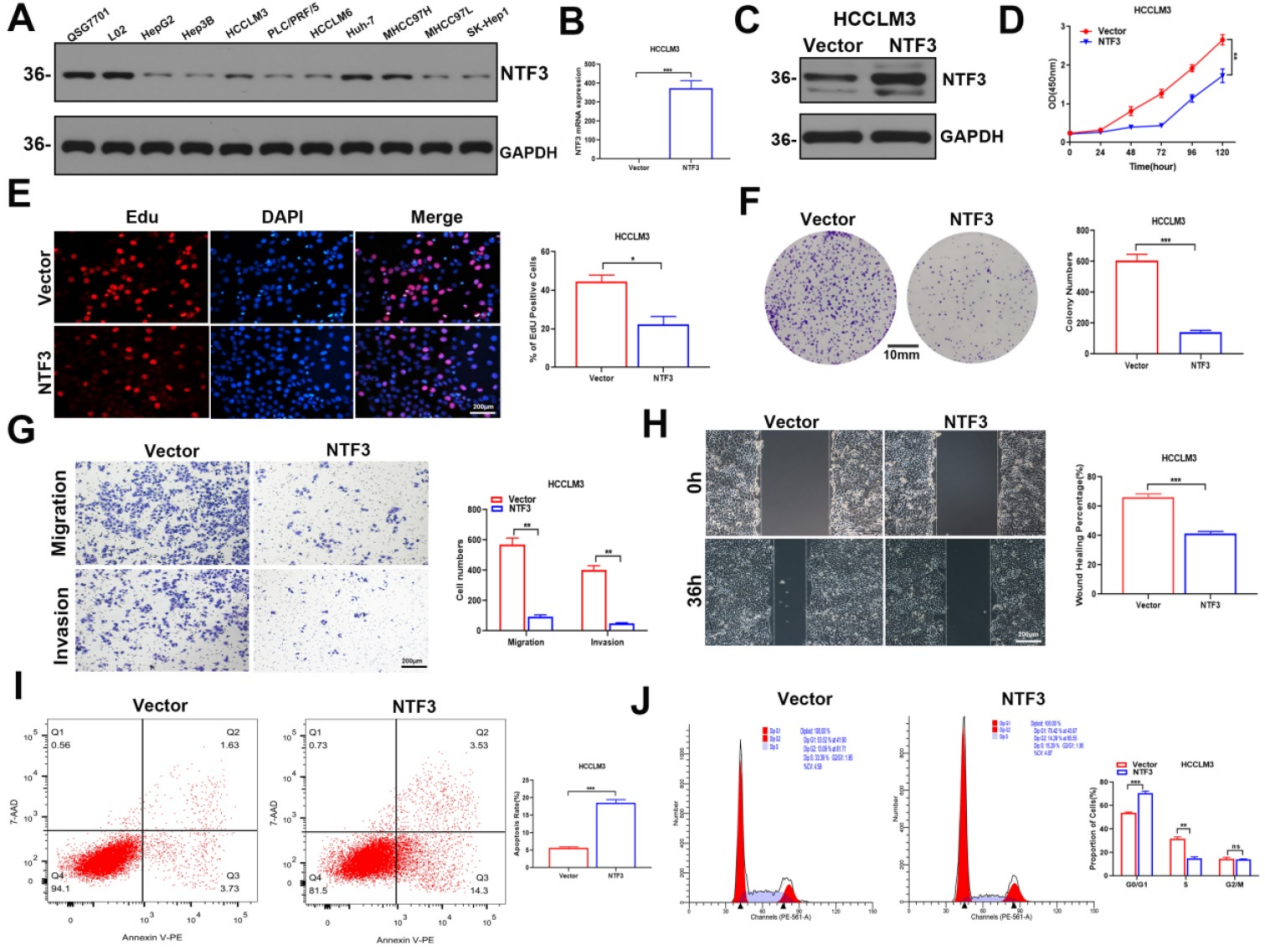
** The overexpression of NTF3 inhibited the proliferation, migration, and invasion of HCC cells *in vitro* and induced apoptosis and cycle arrest. A** NTF3 was expressed in QSG7701, L02, and 9 HCC cell lines. **B, C** qRT-PCR and WB results verified that the stable overexpression of NTF3 in HCCLM3 was successfully constructed. **D, E, F** CCK8, colony formation, and EDU revealed that NTF3 inhibited the proliferation of HCC cells.** G, H** Wound-healing, migration, and invasion experiments indicated that NTF3 inhibited the migration and invasion of HCC cells.** I, J** Flow cytometry detected cell apoptosis and cycle. NTF3 promoted apoptosis, and the cycle was blocked in the G0/G1 phase. *P < 0.05; **P < 0.01; ***P < 0.001.

We found that NTF3 was overexpressed in QSG7701 and L02 but slightly expressed in Huh-7 and MHCC97H. In the remaining cell lines HCCLM3, HCCLM6, Hep3B, PLC/PRF/5, HepG2, SK-Hep1, and MHCC97L, NTF3 expression was weakly (Fig. 2A).

To better determine the antitumor effect of NTF3, we first established stable transfection cell lines by overexpressing NTF3 in HCCLM3 and Huh-7. This successful establishment of stably transfected cell lines was verified by qRT-PCR and WB (Fig. 2B, C; Fig. S1A, S1B). CCK8, EDU, and colony formation experiments revealed that the proliferation ability of HCC cells was weakened after NTF3 overexpression (Fig. 2D-F; Fig. S1C-S1E). Wound healing, migration, and invasion experiments showed that after NTF3 overexpression in HCC cells, their migration and invasion abilities were weakened when compared with those in the control group (Fig. 2G, H; Fig. S1F, S1G). Flow cytometry was used to detect cell cycle and apoptosis. When compared with the control group, the NTF3 overexpression group showed increased cell apoptosis, and the cell cycle was blocked in the G0/G1 phase (Fig. 2I, J; Fig. S1H, S1I).

### NTF3 overexpression inhibits tumor growth and EMT transformation *in vivo*

**Figure 3:**
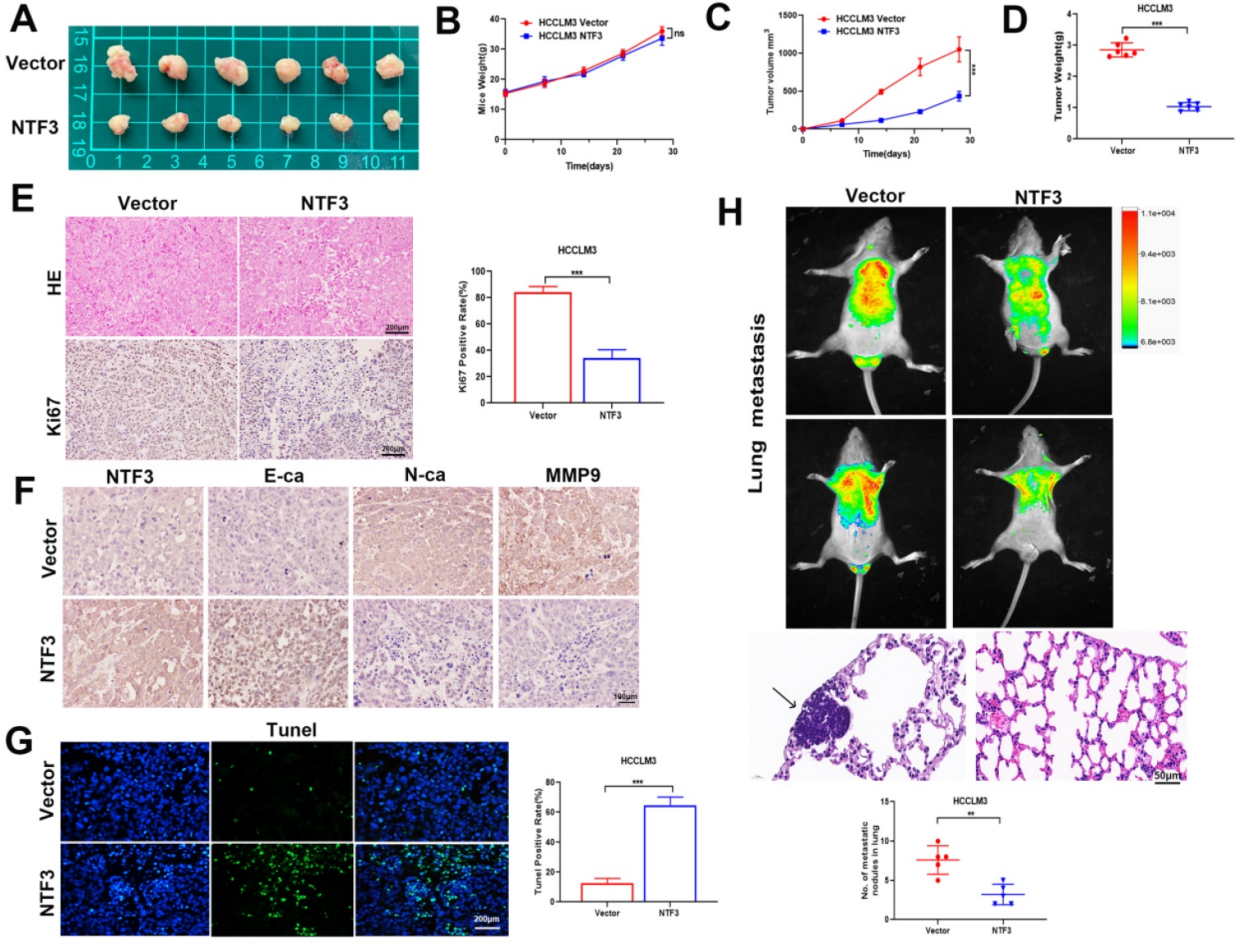
** The overexpression of NTF3 inhibited tumor growth *in vivo* and inhibited EMT transformation. A** HCCLM3 overexpressing NTF3 (n=6) and the control group (n= 6) were implanted into nude mice for subcutaneous tumor imaging.** B** The growth curve of nude mice in the NTF3 group and control group.** C, D** The NTF3 group and control group tumor growth curve and tumor weight. **E** The representative images of hematoxylin-eosin (HE) staining and Ki67 immunostaining tumor tissues. **F** NTF3, E-cadherin (E-ca), N-cadherin (N-ca), and MMP9 immunohistochemical imaging of nude mouse tumor. **G** Tunel imaging of nude mouse tumor. **H** Representative image of HE staining and luciferase images of lung metastatic tumor. *P < 0.05; **P < 0.01; ***P < 0.001.

To identify the underlying antitumor mechanism of NTF3, NTF3-overexpressing cells were injected into the armpit and tail vein of BALB/c nude mice to establish a lung metastasis model. When compared with the weight of the control group, that of the BALB/c nude mice did not change significantly, albeit the tumor weight and volume of the NTF3 overexpressing group had significantly reduced (Fig. 3A-D). Immunohistochemical analysis revealed that NTF3 overexpression inhibited EMT transformation, increased E-cadherin expression, and decreased N-cadherin and Ki67 expression (Fig. 3E, F). The Tunel experiment showed that apoptosis increased after NTF3 overexpression (Fig. 3G). In the mouse model of lung metastasis, NTF3 overexpression caused a significant reduction in the number of lung tumors (Fig. 3H).

### c-Jun promotes NTF3 transcription

**Figure 4:**
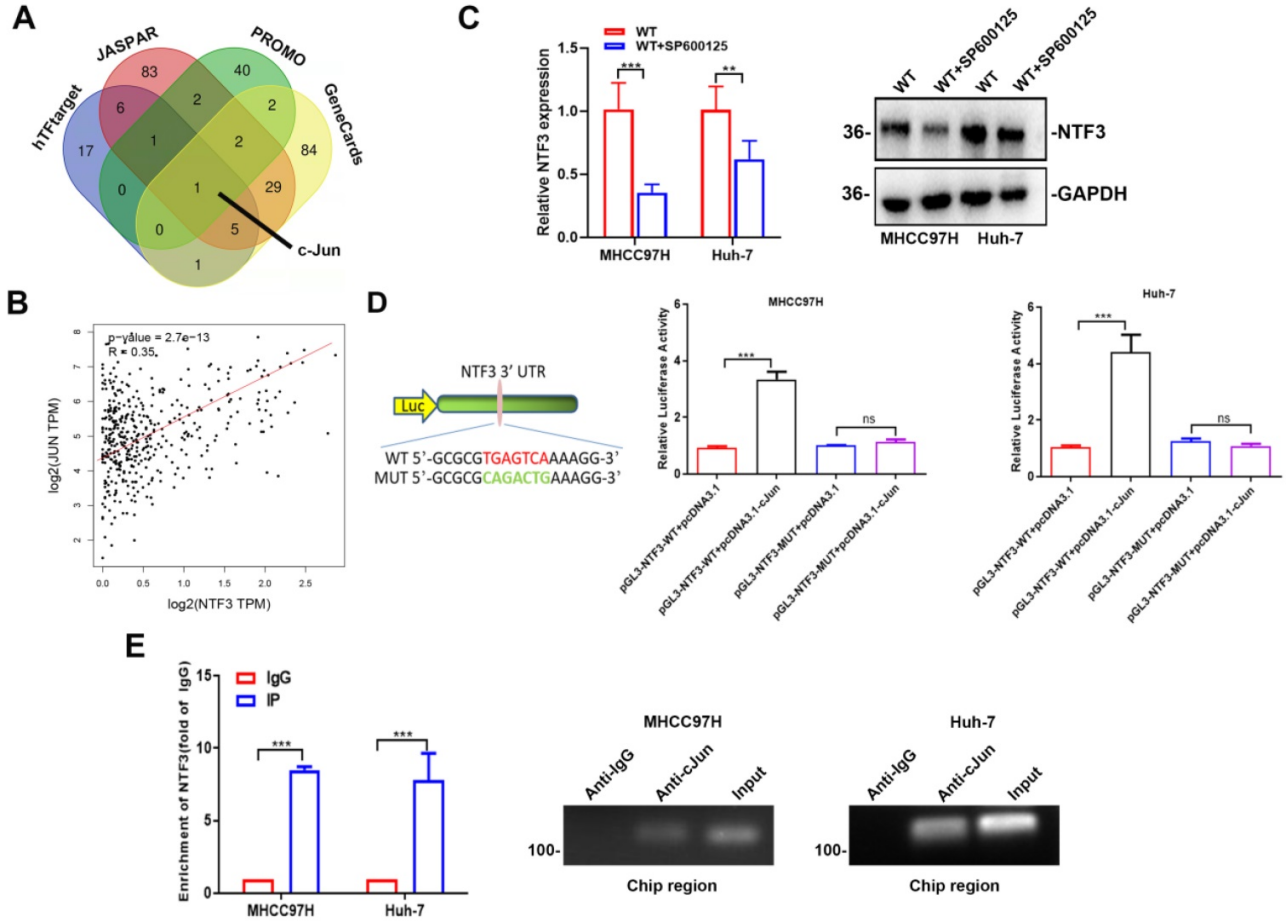
** The transcription level of NTF3 was directly regulated by c-Jun. A** Screening NTF3 upstream transcription factors through 4 databases: JASPAR, PROMO, hTFtarget, and GeneCards. **B** c-Jun and NTF3 as positive feedback by TCGA-LIHC. **C** After SP600125(20 µM) was added to MHCC97H and Huh-7, the expression of NTF3 were decreased. **D** The Luciferase Reporter Assay was performed in MHCC97H and Huh-7. After mutation of c-Jun and the NTF3 binding site, the expression of luciferase was significantly increased. **E** In MHCC97H and Huh-7, ChIP proved that c-Jun binds to the NTF3 promoter region.*P < 0.05; **P < 0.01; ***P < 0.001.

We combined the 4 databases JASPAR, PROMO, hTFtarget,and GeneCards to predict NTF3 transcription factor, and selected the intersection to determine c-Jun as the upstream transcription factor of NTF3 (Fig. 4A). Through TCGA-LIHC database analysis, it was found that c-Jun was positively correlated with NTF3 expression and had a strong correlation (Fig. 4B). As is already known, c-Jun is a downstream factor of JNK; therefore, we added JNK inhibitor SP600125 (20 µM) to MHCC97H and Huh-7, and found that NTF3 expression at both the mRNA and protein levels had decreased (Fig. 4C, D). It was further proved that c-Jun stimulates NTF3 transcription. For further verification, we conducted a luciferase experiment and a ChIP experiment. In the MHCC97H and Huh-7, the binding sites of NTF3 and c-Jun were mutated, and it was found that the expression of luciferase was significantly increased, indicating that the mutation of the binding site had abolished the ability of c-Jun to promote NTF3 transcription (Fig. 4E). In the MHCC97H and Huh-7, ChIP experiments confirmed that c-Jun binds to the NTF3 promoter region. In conclusion, we found that c-Jun could indeed promote NTF3 transcription by binding to the NTF3 promoter region.

### NTF3 is regulated by histone acetylation

Epigenetic regulation can be classified into 3 types: histone methylation, DNA methylation, and histone acetylation 18. Past reports on the sequencing results have suggested that the upstream of NTF3 is enriched to EZH2 19. We, therefore, speculated that the upstream region of NTF3 was related to histone methylation. Accordingly, we added the histone methylation inhibitor 3-deazaneplanocin A hydrochloride (0, 1.25, 2.5, 5, and 10 µM) to Huh-7, cultured the cells for 2 days in a serum-free medium, and detected *NTF3* mRNA expression. No significant changes were observed (Fig. S2A). For detecting DNA methylation, the DNA methylation inhibitor decitabine (0, 10, and 20 µM) was added to Huh-7 and the cells were cultured in a serum-free medium for 2 days.* NTF3* mRNA expression was subsequently detected and no significant changes were observed (Fig. S2B). After verifying histone acetylation, Huh-7 and HCCLM3 were treated with the histone deacetylation drug entinostat (0, 5, and 10 µM) in a serum-free medium. After 2 days of treatment, *NTF3* mRNA and protein levels were significantly upregulated (Fig. S2C, D). Entinostat is a histone deacetylation drug and HDAC1/HDAC3 are its targets. To verify the specific target, Huh-7 and HCCLM3 were treated with varying concentrations of romidepsin (0, 25, 50, and 100 µM of romidepsin for Huh-7 and 0, 5, and 10 µM of romidepsin for HCCLM3) and the target was HDAC1/2. RGFP966, whose target was HDAC3 acted on Huh-7 and HCCLM3 at concentrations of 0, 12.5, and 25 µM in a serum-free medium for 2 days. As shown in Figures S2E and S2F, romidepsin did not cause any significant changes in NTF3 mRNA expression. However, NTF3 mRNA expression increased significantly after RGFP966 treatment (Fig. S2G). These results implied that HDAC3 plays a major role in NTF3 regulation. For confirming this finding, we used HDAC3 small interference RNA to knockdown Huh-7 and HCCLM3 cells and found that the mRNA and protein levels of NTF3 had increased significantly in these cells (Fig. S2H, I). Therefore, we concluded that the upstream region of NTF3 was regulated by histone deacetylation by HDAC3. Please refer to Supplementary Material Table S5 for the details regarding the drugs used in this study.

### NTF3 binds to p75NTR and promotes apoptosis through the apoptotic signaling pathway

**Figure 5:**
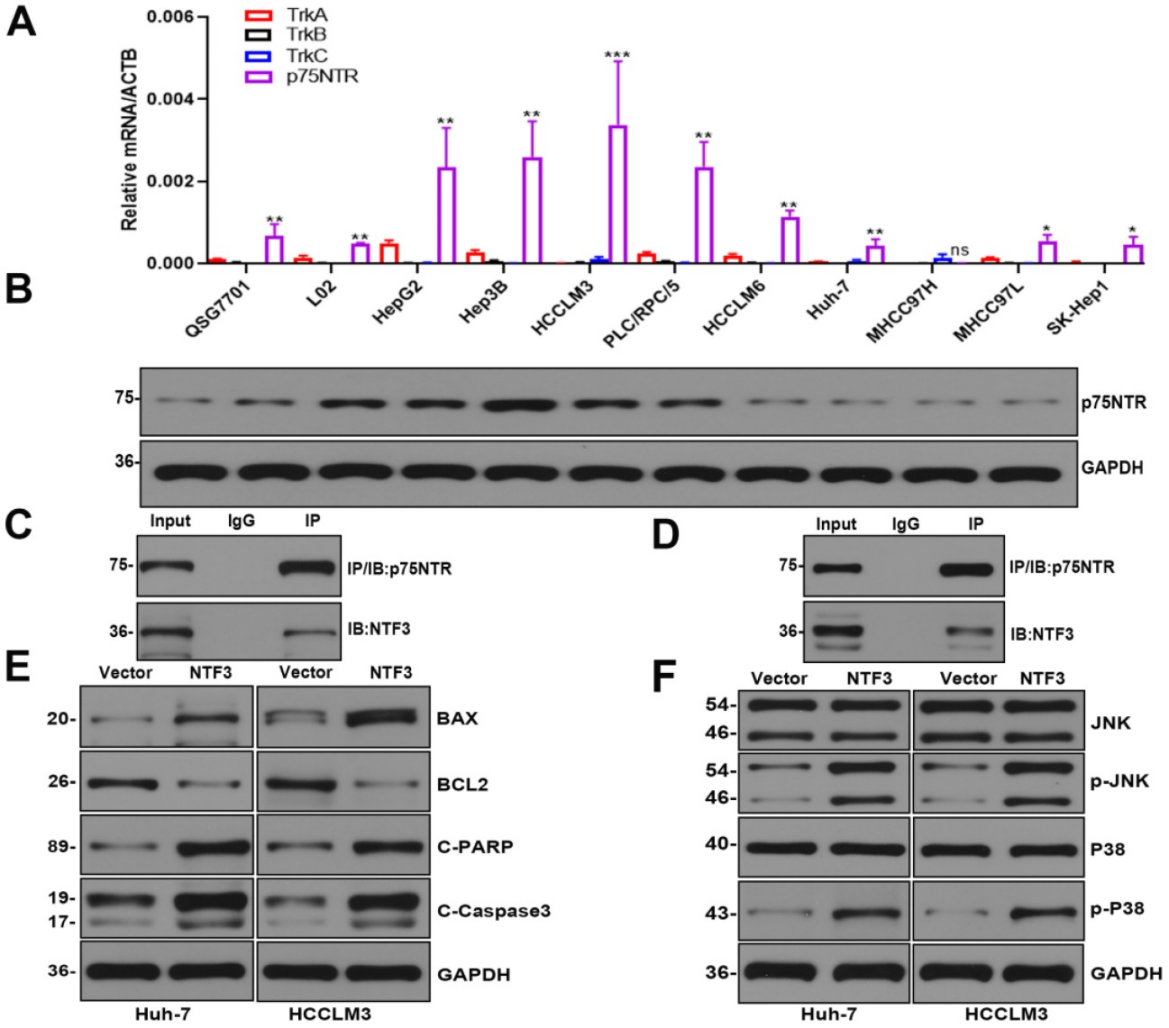
** NTF3 acted as a ligand and bound to the receptor p75NTR to promote cell apoptosis. A** qRT-PCR verified the expression of TrkA/B/C mRNA and p75NTR mRNA in QSG7701, L02, and 9 HCC cell lines. **B** WB verified the expression of p75NTR in QSG7701, L02, and 9 HCC cell lines. **C, D** Endogenous co-IP of Huh-7 and HCCLM3 revealed that NTF3 interacts with p75NTR.** E** The NTF3 and the control group of HCCLM3 and Huh-7 apoptosis-related protein levels changed. **F** NTF3 and the control group of HCCLM3 and Huh-7 were used to detect changes in the total phosphorylated JNK and P38 levels. *P < 0.05; **P < 0.01; ***P < 0.001.

Past studies have reported that NTF3, as an NT, has two most common receptors: the tropomyosin tyrosine kinase receptor (Trk) and the common NT receptor p75NTR 9,10. We verified that TrkA/TrkB/TrkC was not expressed in HCC. The level of the p75NTR receptor protein encoded by the NGF receptor (NGFR) was detected in HCC by qRT-PCR and Western blotting experiments (Fig. 5A,B). The endogenous Co-IP of Huh-7 and HCCLM3 indicated that NTF3 can bind to the p75NTR receptor (Fig. 5C, D).

Considering that the experimental results showed that NTF3 has a significant pro-apoptotic function and interaction with p75NTR, we attempted to identify its potential signaling pathways. We first detected apoptosis-related proteins in HCCLM3 and Huh-7 cells overexpressing NTF3 and found that when compared with the control group, the expression of BCL2 was downregulated, while that of BAX, Cl-caspase3, and Cl-PARP was upregulated, which further confirmed the stimulatory effect of NTF3 on apoptosis (Fig. 5E). To verify the potential signaling pathways in which NTF3 is involved, we detected JNK and P38, and their phosphorylated levels indicated that the expression of phosphorylated p-JNK and p-P38 increased significantly (Fig. 5F). Moreover, we also considered the results of a past study on the effect of p75NTR on apoptosis to confirm our results 11. Accordingly, we concluded that NTF3 acts as a ligand and binds to the p75NTR receptor and promotes apoptosis through the JNK and P38 MAPK pathways.

### Verification of NTF3 binding to p75NTR

**Figure 6:**
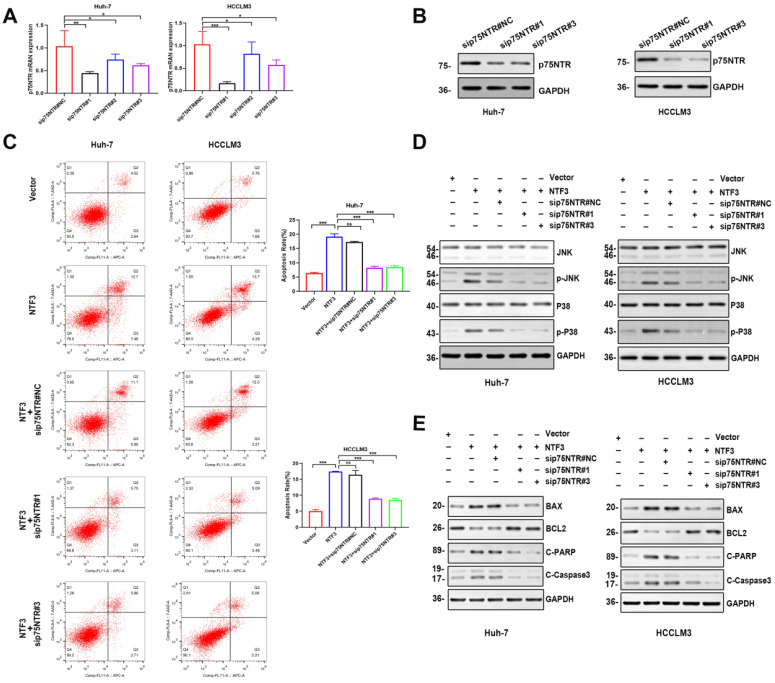
** NTF3 and p75NTR are bound to each other. A,B** qRT-PCR and WB detection of p75NTR knockdown efficiency in HCCLM3 and Huh-7. **C** Sets of 5 groups of 1) NTF3#NC, 2) NTF3, 3) NTF3+sip75NTR#NC, 4) NTF3+siNGRF#1, 5) NTF3+siNGRF#3 in HCCLM3 and Huh-7, and the detection of changes in apoptosis by flow cytometry. **D** WB detected the total and phosphorylated JNK and P38 level changes. **E** WB detected changes in the apoptosis-related protein levels. *P < 0.05; **P < 0.01; ***P < 0.001.

To confirm whether NTF3 exerted a pro-apoptotic effect after binding to p75NTR, we prepared small interference RNA of p75NTR. First, PCR and WB analyses were performed to verify that the knockdown efficiency of sip75NTR#1 and sip75NTR#3 (Fig. 6A, B). Huh-7 and HCCLM3 were further divided into 5 groups (NTF3#NC, NTF3, NTF3+sip75NTR#NC, NTF3+sip75NTR#1 and NTF3+sip75NTR#3), and the apoptotic changes in each group were confirmed by flow cytometry (Fig. 6C). We also detected the changes and expression of JNK and P38. We found that, when compared with that in the control group, after knocking down p75NTR, the expression of p-JNK and p-P38 was downregulated (Fig. 6D), whereas the expression of BCL2 was upregulated, while that of BAX, Cl-caspase3, and Cl-PARP was downregulated (Fig. 6E). These results confirmed that NTF3 played a role in promoting apoptosis by binding to p75NTR.

Interestingly, we found that entinostat (target of HDAC1/HDAC3) acts as a tumor suppressor and a pro-apoptosis and cell cycle-blocking drug and acts on HCCLM3 and Huh-7 (concentrations of 0, 5, and 10 µM). Both these cell types were cultured in a serum-free medium for 2 days, and we found that the expression of p75NTR had increased (Fig. S3A, S2B). To verify the specific target, romidepsin (target is HDAC1/2) was added to Huh-7 at concentrations of 0, 25, 50, and 100 µM and to HCCLM3 at concentrations of 0, 5, and 10 µM in the serum-free medium, and the cells were cultured for 2 days. qRT-PCR analysis revealed that the expression of p75NTR was regulated mainly because of HDAC1 (Fig. S3C). After knocking down HDAC1 in Huh-7 and HCCLM3, we found that p75NTR mRNA and the protein levels decreased significantly (Fig. S3D, S2E). Entinostat can also upregulate the expression of NTF3 and p75NTR, and activate the NTF3/p75NTR pathway.

### JNK inhibitor SP600125 and P38 inhibitor SB20358 reversed the antitumor effect induced by NTF3

**Figure 7:**
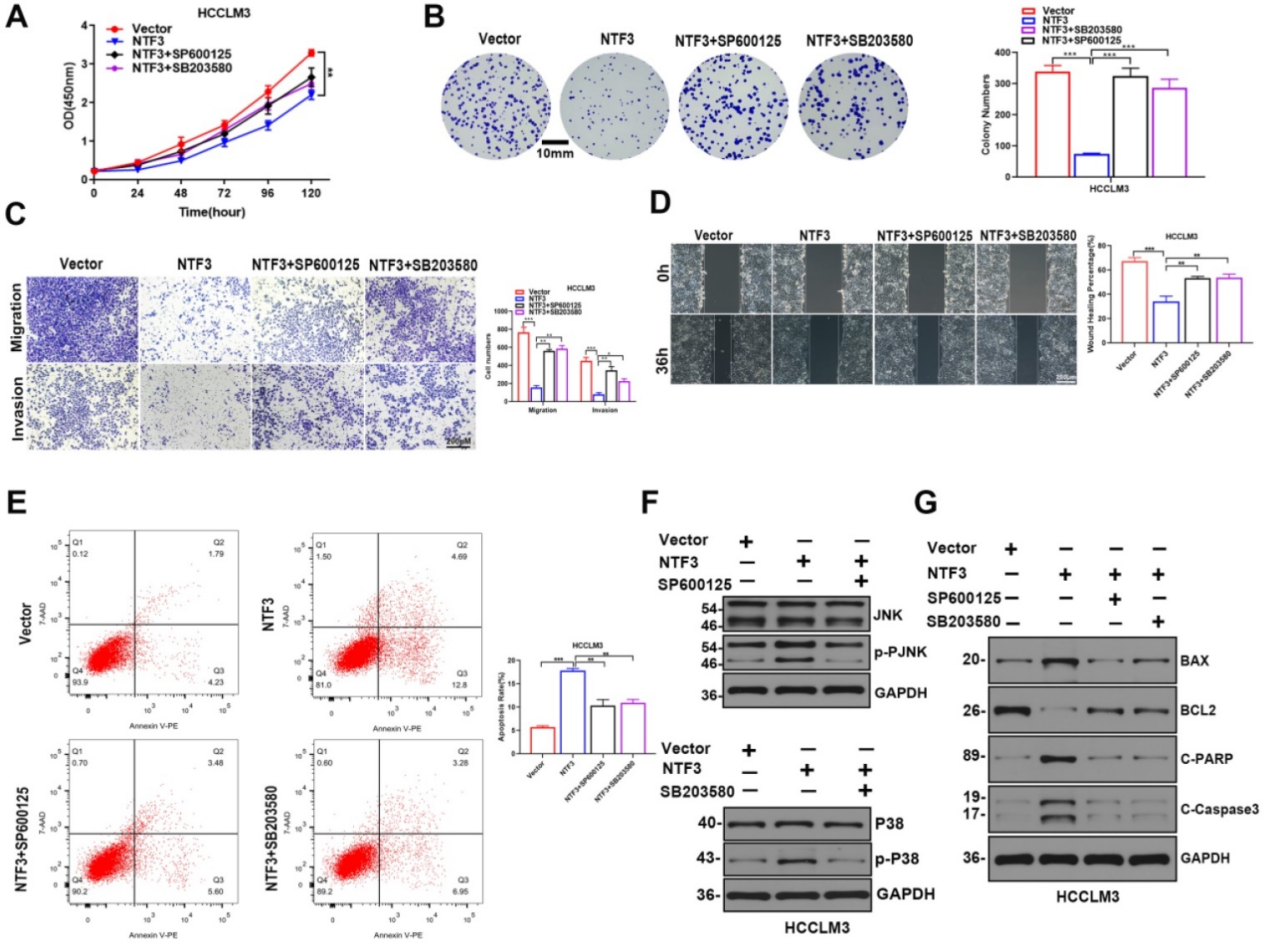
** JNK inhibitor SP600125 and P38 inhibitor SB20358 reversed the anti-tumor effect induced by NTF3. A, B** CCK8 and colony formation experiments suggested that the addition of inhibitors reversed the inhibitory effect of NTF3-induced proliferation. **C, D** Wound-healing, invasion, and migration experiments suggested that the addition of inhibitors reversed the invasion and migration effects of NTF3. **E** Set of 4 groups in HCCLM3: 1) Vector, 2) NTF3 group, 3) NTF3+SP600125, 4) NTF3+SB20358; the changes in apoptosis were detected by flow cytometry.** F** WB detected the changes in the total and phosphorylated JNK and P38 levels. **G** WB detected the changes in the apoptosis-related proteins in the 4 groups. *P < 0.05; **P < 0.01; ***P < 0.001.

Our results revealed that the mutual action of NTF3 and p75NTR synergistically induced apoptosis in HCC cells and a previous study showed that p75NTR promotes apoptosis through the JNK and P38 pathways 11. To verify whether the JNK and P38 pathways promote apoptosis in HCC cells, we divided Huh-7 and HCCLM3 cells into the following 4 groups: vector: NTF3-overexpressing negative control group; NTF3: NTF3-overexpressing group; NTF3+SP600125, and NTF3+SB20358. These groups were prepared as follows: after starving for 12 h when the cell density was approximately 40%, the high-sugar DMEM medium was replaced with 1% FBS and then the JNK pathway inhibitor SP600125 (20 µM) and the P38 MAPK pathway inhibitor SB20358 (40 µM) were added followed by culturing for 2 days. For detecting the corresponding cell function and apoptosis-related proteins, we found that, when compared with the NTF3 group, the NTF3 group with SP600125 and SB20358 demonstrated significantly increased proliferation (Fig. 7A, B; Fig. S4A, S4B). The addition of inhibitors reversed the invasion and migration ability of NTF3 inhibition (Fig. 7C, D; Fig. S4C, S4D). Flow cytometry indicated that NTF3-induced apoptosis was partially restored after the inhibitors were added (Fig. 7E; Fig. S4E). Western blotting showed that, after the addition of SP600125 and SB20358 to HCCLM3 and Huh-7 cells, NTF3-induced apoptosis-related protein expression was reversed (Fig. 7F; Fig. S4F) and that led to changes in the JNK and P38 expression (Fig. 7G; Fig. S4G). These results collectively indicate that NTF3 and p75NTR bind to each other and induce HCC cell apoptosis by promoting JNK and P38 activation.

### Knockdown NTF3 promotes HCC progression

**Figure 8:**
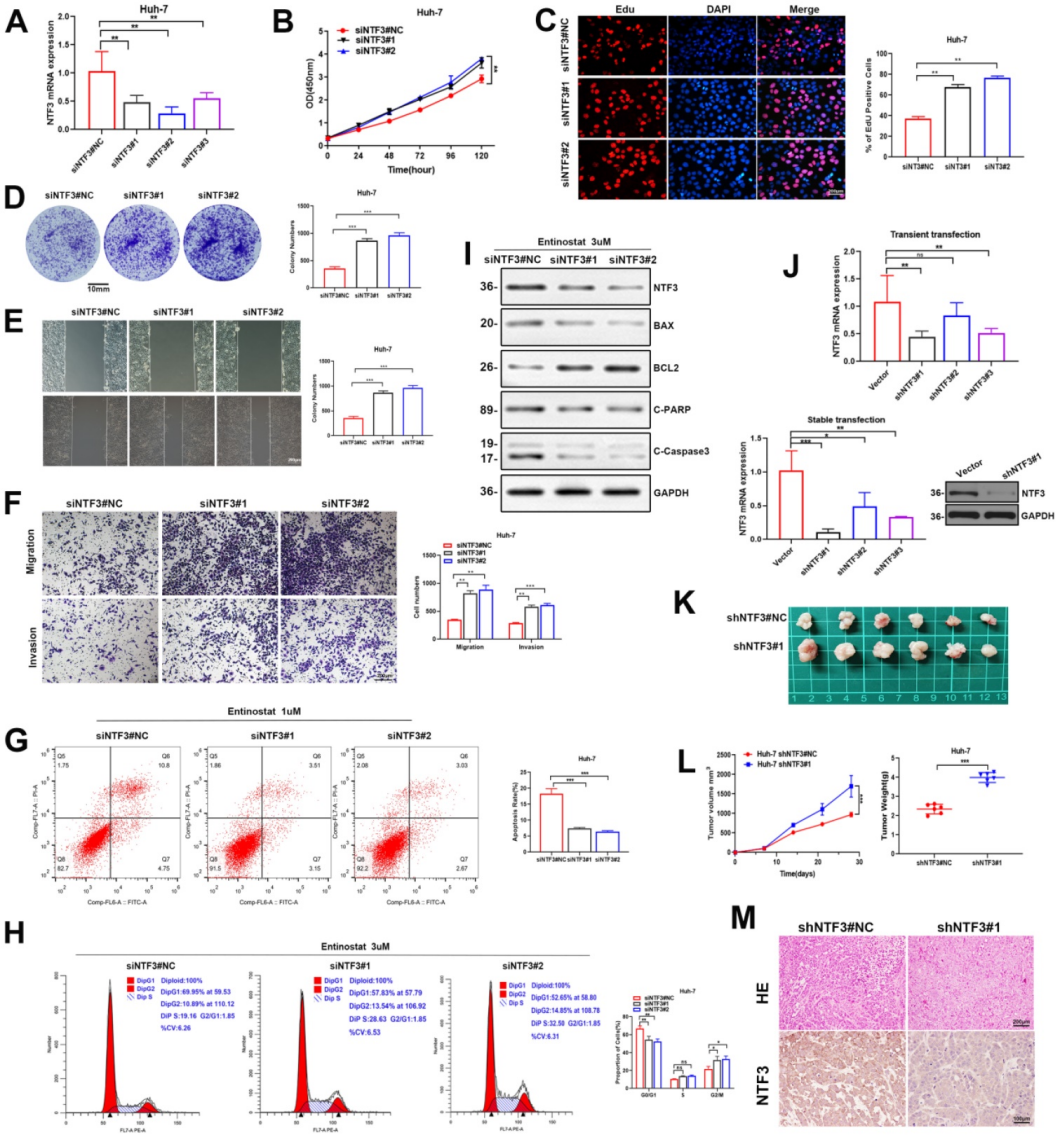
** Knockdown of NTF3 promotes HCC cells proliferation* in vitro* and *in vivo*, increases migration and invasion, and decreases apoptosis. A** The knockdown efficiency of *NTF3* mRNA was verified. **B, C, D** After knocking down NTF3, CCK8, and EDU, the colony formation experiments suggested that it promoted cell growth. **E, F** Wound-healing, invasion, and migration experiments suggested that the knockdown of NTF3 increased the invasion and migration ability. **G,H** Huh-7 were added to Entinostat, and flow cytometry was performed to indicate that the cycle was downregulated in the G0/G1 phase and that the apoptosis was decreased after NTF3 knockdown. **I** After the knockdown of NTF3, WB detected changes in the levels of apoptosis-related proteins. **J** PCR and WB verified that the NTF3-stable transgenic virus strain was screened out in Huh-7. **K** Huh-7 knocking down NTF3 and control group were implanted into nude mice for subcutaneous tumor imaging. **L** After the knockdown of NTF3, the volume and weight of the animal subcutaneous tumors increased significantly. **M** Representative images of tumor tissues with hematoxylin-eosin (HE) staining and NTF3 immunostaining. *P < 0.05; **P < 0.01; ***P < 0.001.

To further confirm the effect of NTF3 on cell function, after knocking down NTF3 via small interference RNA, qRT-PCR revealed that the knockdown efficiency of siNTF3#1 and siNTF3#2 were better (Fig. 8A). CCK8, EDU, and colony-formation experiments showed that the proliferation ability was enhanced after knocking down NTF3 when compared with the control group (Fig. 8B-D). Wound healing, migration, and invasion experiments showed that the cell invasion ability was increased after knocking down NTF3 when compared with the control group (Fig. 8E, F). Considering that after knocking down NTF3, the apoptotic rate may decrease, we added entinostat (1 µM) to increase the basal apoptotic rate, after which, apoptosis was detected by flow cytometry. When compared with the control group, the apoptosis of the NTF3 knocked down group was reduced. Entinostat (3 µM) was added to detect the cell cycle by flow cytometry, and when compared with the control group, the cells in the NTF3 knockdown group were found to be arrested in the G0/G1 phase (Fig. 8G, H). The detection of apoptosis-related proteins indicated that, after the addition of 3 µM entinostat, when compared with the control group, the expression of NTF3, BAX, Cl-PARP, and Cl-caspase3 was downregulated, whereas that of BCL2 was upregulated, which further confirmed the effect of NTF3 on apoptosis (Fig. 8I). Lentivirus-mediated NTF3 targeting short-hairpin RNA (shRNA) was used to transfect the Huh-7 cells (MOI=5), and the screen effective knockdown of cell lines revealed an efficiency of approximately 85% (Fig. 8J). Knockdown stably transformed Huh-7 was injected subcutaneously (6×10^6^ cells) into the armpit of male BALB/c nude mice. When compared with the control group, the tumor weight and volume in the experimental group showed a significant increase (Fig. 8K, L). Immunohistochemical analysis revealed that the NTF3 knockdown decreased the NTF3 expression (Fig. 8M). *In vivo* experiments further verified that NTF3 is a tumor suppressor.

## Discussion

Research on other molecules belonging to the NT family, such as brain-derived neurotrophic factor BDNF, acted as a potent angiogenic factor, revealing that these molecules stimulate BNL cells by upregulating the TrkB expression, and inducing new blood vessel formation through the VEGF pathway, which ultimately promotes tumor growth 21. In patients with CRC, the high serum levels of BDNF and NT4/5 cannot be considered markers of disease progression or tumor stage, but rather can be used as indicators of good prognosis 22. Targeting the BDNF/TrkB pathway can also prevent or inhibit epilepsy. NTs have been widely used as biomarkers and especially BDNF in neurological diseases 23. The TrkB receptor agonist R13 mimics the effect of BDNF and effectively improves the exercise capacity of ALS mice by improving mitochondrial function 24.

Kim et al. 12 and Kim et al. 13 found that the NTF3/TrkC signal has a significant antitumor effect on MBL cells. In primitive neuroectodermal brain tumors (PNETS), TrkC accelerates apoptosis and terminal neuronal differentiation through its ligand NTF3 14,15. Louie et al. 16 found that NTF3 promotes the growth of brain metastasis tumor cells derived from breast cancer. Neurotrophic factor 3 (NTF3) (a key mediator of neuronal development during early neurogenesis) is a putative regulatory target of POU3F2, and human neuron differentiation and survival are controlled by the POU3F2/NTF3 pathway 25.

In this study, we found that NTF3 was significantly downregulated in HCC and related to poor prognosis. NTF3 inhibits HCC cell proliferation, invasion, and migration and promotes apoptosis *in vitro* and *in vivo*. c-Jun or c-Fos induce apoptosis in various cell lines 26. c-Jun is a common transcription factor. As a component of AP-1, it induces the activation of various genes, such as FasL, Bim, or Bcl3, and its product is a positive regulator of apoptosis 27. In different cell types, the balance between pro-apoptotic and anti-apoptotic target genes determines whether the final result of the cell is survival or death 28. In this study, we determined that c-Jun can promote NTF3 transcription to a certain extent through multi-database joint analyses. These findings provide new biological insights into the c-Jun signaling pathway. Meanwhile, c-Jun, acted as the downstream of the JNK signaling pathway 29, which forms c-Jun/NTF3/JNK/c-Jun positive feedback.

Considering NTF3 acts as a tumor-suppressor gene, the most common way to regulate tumor suppressor genes is epigenetics, which is divided into 3 categories: histone methylation, DNA methylation, and histone acetylation. We verified that HDAC3 plays a major role in regulating NTF3 expression through histone deacetylation.

The NTF3 receptor is classified into two categories: tropomyosin tyrosine kinase receptor (Trk) and the common NT receptor p75NTR 9,10. A past study reported that NTF3 is under-expressed in HCC, and it is predicted that NTF3 exerts a tumor-suppressive effect via TrkC 17. However, we verified that the TrkA/TrkB/TrkC receptors were not expressed in HCC, whereas p75NTR was expressed in the HCC cell lines. Furthermore, a past study revealed that p75NTR can directly interacted with NTF3 20.

In addition, we verified that NTF3 acts as a ligand and binds to the p75NTR receptor by endogenous Co-IP experiments. p75NTR is a pro-apoptotic protein, and its pathway of inducing apoptosis is special, albeit not similar to the apoptosis signaling pathway commonly activated by other molecules in the TNF receptor superfamily 11. Interestingly, we found that entinostat could regulate p75NTR to increase its expression through HDAC1 18. Moreover, histone deacetylation activated the NTF3/p75NTR pathway.

p75NTR-induced apoptosis is related to the accumulation of cytoplasmic cytochrome C and the specific activation of caspase3;moreover, it promotes cell apoptosis through the JNK and P38 MAPK pathways 11. Based on past cell function experiments and apoptosis assays, we speculated that NTF3 may activate the JNK and P38 MAPK cell apoptosis pathway to inhibit the occurrence and development of HCC. In mammals, the mitogen-activated protein kinase (MAPK) signaling pathway has been classified into 3 major categories: c-junNH2 terminal kinase (JNK), extracellular signal-regulated kinase (ERK), and p38 MAPK. Past studies showed that the regulation of MAPK for cell survival or apoptosis depends on the cell type and the type of stimulus 30-32. We also found that NTF3/p75NTR was related to the JNK and P38 MAPK pathways and that the inhibitors reversed the inhibitory effect on HCC.

Our study has some limitations. For example, the number of clinical samples was insufficient (only 74 cases), which did not fully explain the effect of NTF3 on tumor staging, tumor size, liver cirrhosis, lymph node metastasis and other clinicopathological indicators. We did not develop a PDX model (Patient-derived Xenograft. A tumor model was generated via implantation of fresh human tissues into immunodeficient mice). PDX can be used as an important tool to study tumor biology or simulate tumor drugs. It can simulate an *in vivo* environment very well (due to immunodeficiency, it has certain differences in environmental simulation),and because it is directly derived from the cells in a patient, the conclusions drawn on PDX can be used to guide the diagnosis and treatment of the patient, avoiding individual differences caused by tumor heterogeneity as much as possible. In the future, we can foresee that PDX (and of course various other types of tumor models, such as cell lines and organoids) will provide more in-depth guidance and assistance for tumor-related research 33. We did not design any truncation mutant of p75NTR, and after designing the truncation mutant, the specific region that binds to NTF3 was further investigated by immunoprecipitation.

Combination therapy represents a new, more global strategy to augment the possible benefits of combining the existing disease-modifying therapies or other new drugs with acceptable rationale and favorable safety and efficacy 34. According to our study results, NTF3 can combine with p75NTR to promote apoptosis through JNK and P38 MAPK pathways, thereby inhibiting the occurrence and development of HCC. NTF3 is considered a tumor suppressor. Many of the effects of p75NTR on cancer cells are inhibitory due to its direct action 35. Therefore, we considered the combination therapy of NTF3 agonist and p75NTR agonist, which can be verified via animal experiments to enhance the NTF3 activity *in vivo* and can have a good prognosis for animals with liver tumors. As a protein ligand, NTF3 pays special attention to the binding process of ligand and protein in drug design, and we, therefore, expect to develop drugs targeting NTF3 for the treatment of HCC in the future.

This study found that NTF3 could inhibit the occurrence and development of HCC, and through TCGA, GEO, GEPIA, LIHC, and other databases as well as the clinical data collected at our center, it was established that patients with a high expression of NTF3 showed better prognosis and longer OS. Patients with low expression of NTF3 showed a poor prognosis but possibly benefited from the combined treatment with NTF3 and p75NTR.

**Figure 9:**
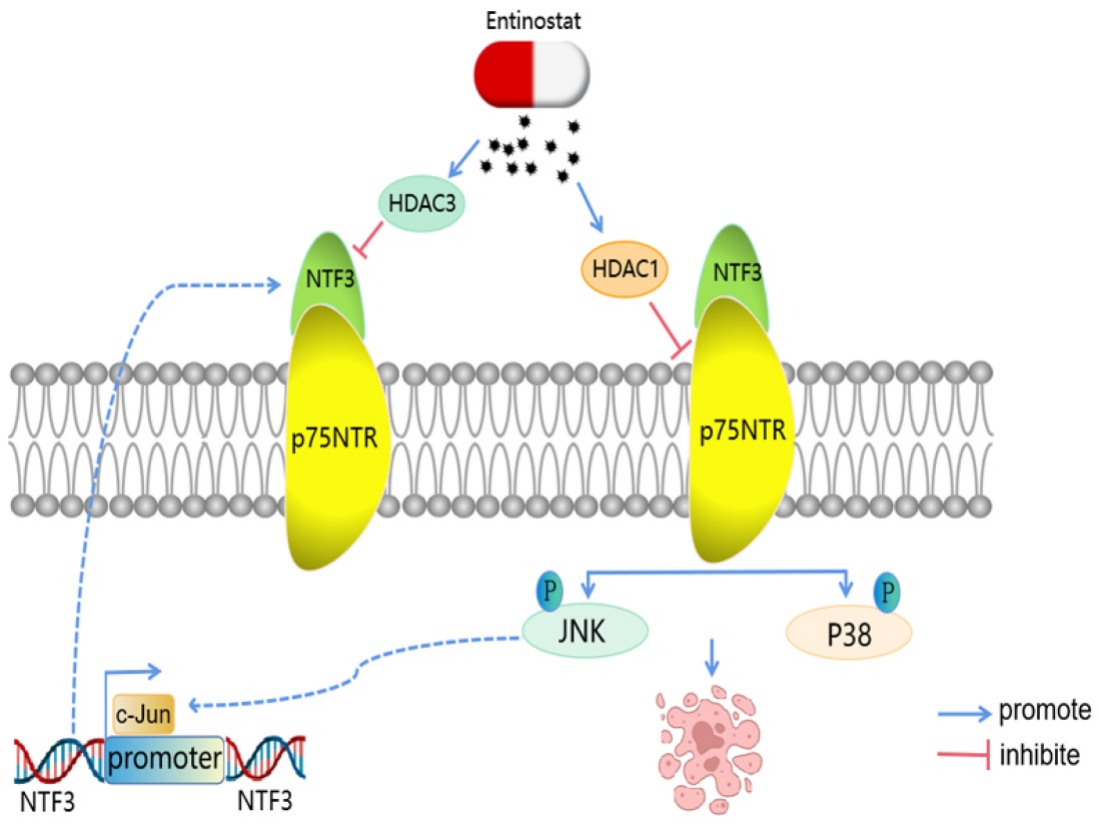
** A model of the regulatory mechanisms of NTF3 in HCC.** c-Jun promotes NTF3 transcription.Entinostat can control the NTF3/p75NTR pathway. NTF3 interacted with p75NTR to facilitate the JNK/P38 MAPK and promote cell apoptosis in HCC.

In summary, we have found that NTF3 is an anti-tumor protein that inhibits tumor proliferation, invasion, and migration through the apoptotic pathway. NTF3 acts as a ligand in HCC and binds to p75NTR, which affects the JNK and P38 MAPK pathway and promotes apoptosis, thereby inhibiting the development of HCC (Fig. 9). Our findings can thus provide a new target for the treatment and prognosis of HCC.

## Supplementary Material

Supplementary figures and tables.

## Abbreviations

HCC: Hepatocellular Carcinoma
NTF3: Neurotrophin3
qRT-PCR: Real-time quantitative polymerase chain reaction
JNK: c-Jun NH2-terminal kinase
TCGA: The Cancer Genome Atlas
GEO: Gene Expression Omnibus
GSEA: Gene set enrichment analysis
HE: Hematoxylin-eosin
IHC: Immunohistochemistry
LIHC: Liver Hepatocellular Carcinoma
SDS-PAGE: Sodium dodecyl sulfate-poly-acrylamide
PVDF: Polyvinylidene difluoride
shRNA NC: Scrambled shRNA
siRNA: Small interfering RNAs
p75NTR: P75 neurotrophin receptor
NGFR: Nerve growth factor receptor
CCK8: Cell counting kit-8
EMT: Epithelial-Mesenchymal Transition
