# Supplementary material for: Neurotrophin3 promotes hepatocellular carcinoma apoptosis through the JNK and P38 MAPK pathways
Source: Int J Biol Sci. 2022 Oct 3;18(15):5963–77. doi: 10.7150/ijbs.72982 (PMC9576519; doi:10.7150/ijbs.72982)
Supplement: Supplementary file 1 — Supplementary figures and tables. [file ijbsv18p5963s1.pdf]

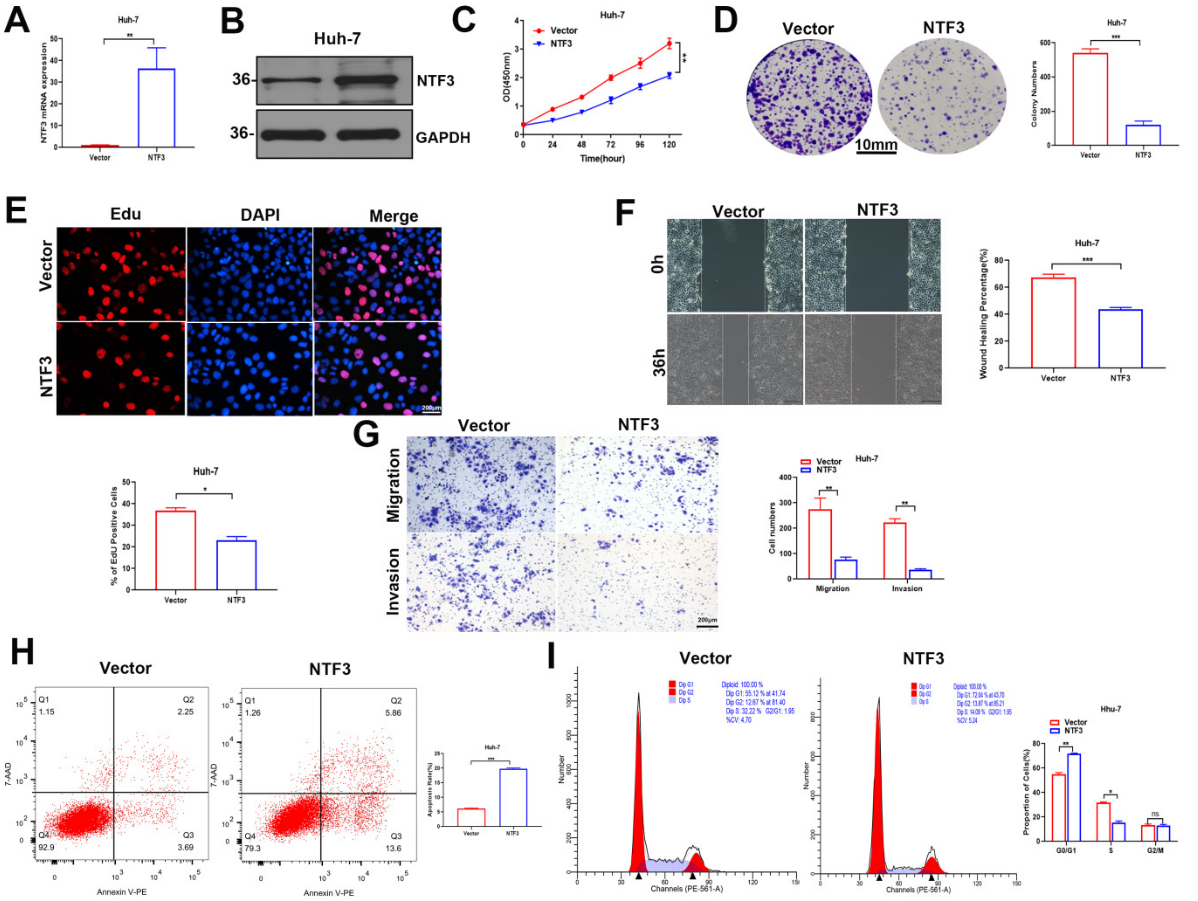

Fig. S1 The overexpression of NTF3 inhibited the proliferation, migration, and invasion of HCC cells *in vitro* and induced apoptosis and cycle arrest.

A,B qRT-PCR, and WB verified that the stable overexpression of NTF3 in Huh-7 was successfully constructed. **C,D,E** CCK8, colony formation, and EDU revealed that NTF3 inhibited the proliferation of HCC cells. **F,G** Wound-healing, migration, and invasion experiments indicated that NTF3 inhibited the migration and invasion of HCC cells. **H,I** Flow cytometry detected cell apoptosis and cycle. NTF3 promoted apoptosis, and the cycle was blocked in the G0/G1 phase. *$P < 0.05$; **$P < 0.01$; ***$P < 0.001$.

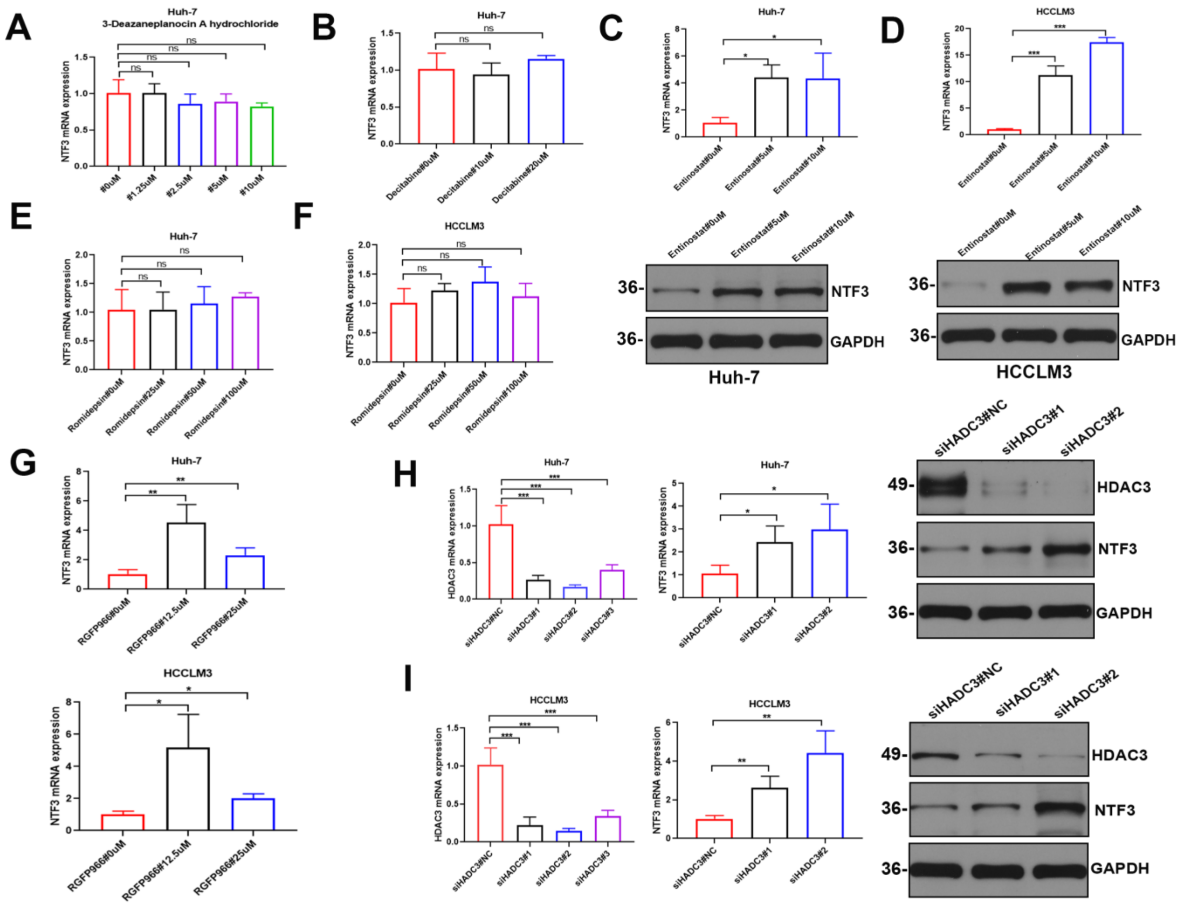

Fig. S2 NTF3 is regulated by histone acetylation.

A Huh-7 was added to the histone methylation inhibitor 3-Deazaneplanocin A hydrochloride (concentrations 0, 1.25, 2.5, 5, and 10 μM) serum-free medium cultured for 2 days, and NTF3 was detected by qRT-PCR. **B** Huh-7 were added to the DNA methylation inhibitor decitabine (concentrations 0, 10, and 20 μM) serum-free medium cultured for 2 days, NTF3 was detected by qRT-PCR. **C, D** Huh-7 and HCCLM3 were added with histone deacetylation drug Entinostat (concentration 0, 5, and 10 μM) serum-free medium cultured for 2 days, and NTF3 was detected by qRT-PCR and WB. **E, F** Romidepsin was added to Huh-7 (concentrations 0, 25, 50, and 100 μM) and HCCLM3 (concentrations 0, 5, and 10 μM, respectively). After 2 days of culturing in a serum-free medium, NTF3 was detected by qRT-PCR. **G** RGFP966 was added to Huh-7 and HCCLM3 (concentrations 0, 12.5, and 25 μM) and cultured in a serum-free medium for 2 days, followed by qRT-PCR detection of NTF3. **H, I** Knockdown of HDAC3 in Huh-7 and HCCLM3 and detection of the expression of HDAC3 and NTF3 by qRT-PCR and WB. *P < 0.05; **P < 0.01; ***P < 0.001.

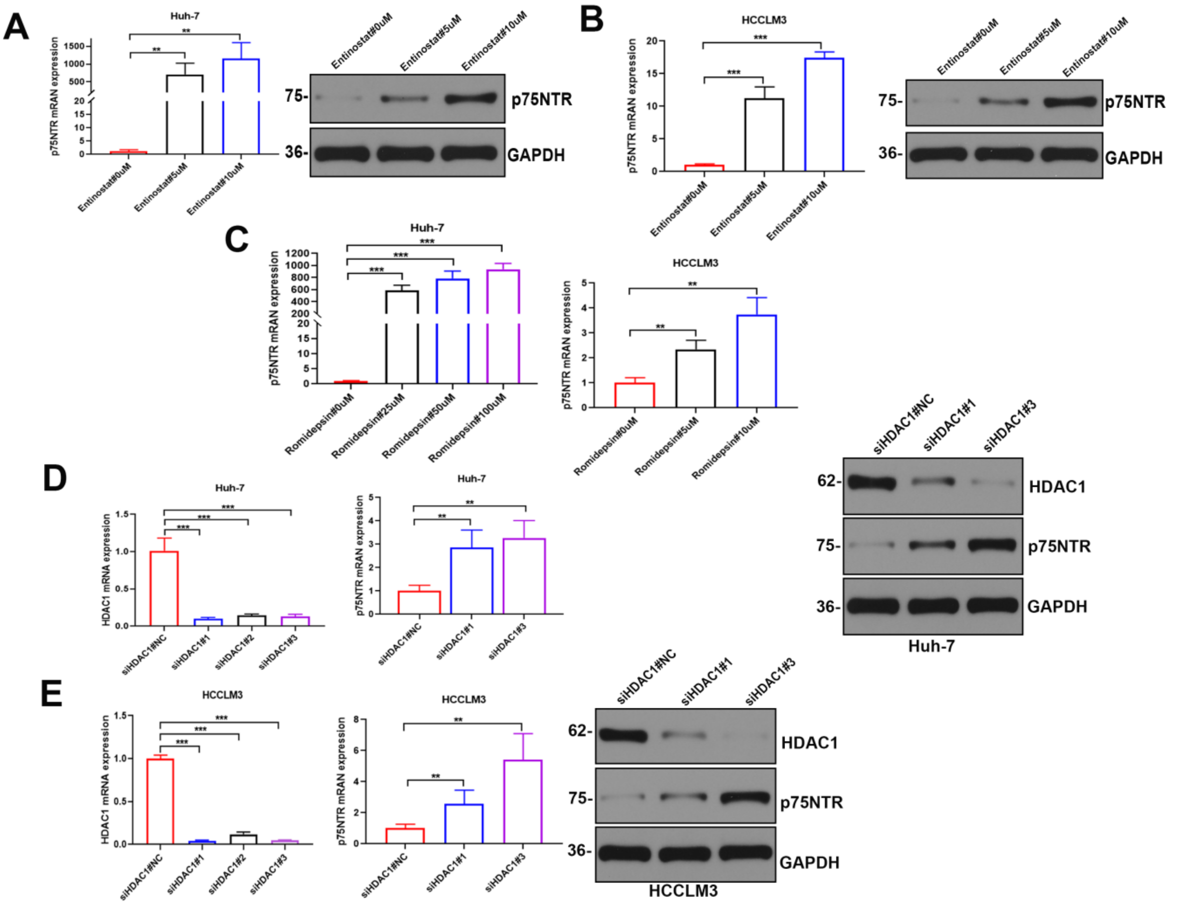

Fig.S3 p75NTR was regulated by histone acetylation.

A, B Huh-7 and HCCLM3 were added to the histone deacetylation drug entinostat (concentrations 0, 5, and 10 μM) in a serum-free medium for culturing after 2 days, followed by qRT-PCR and WB detection of p75NTR. **C** Romidepsin was added to Huh-7 (concentrations 0, 25, 50, and 100 μM) and HCCLM3 (concentrations 0, 5, and 10 μM, respectively) in a serum-free medium for culturing for 2 days, followed by qRT-PCR detection of p75NTR. **D, E** Huh-7 and HCCLM3 knockdown HDAC1, and the expression of HDAC1 and p75NTR was detected by qRT-PCR and WB. **P*<0.05; ***P*<0.01; ****P*< 0.001.

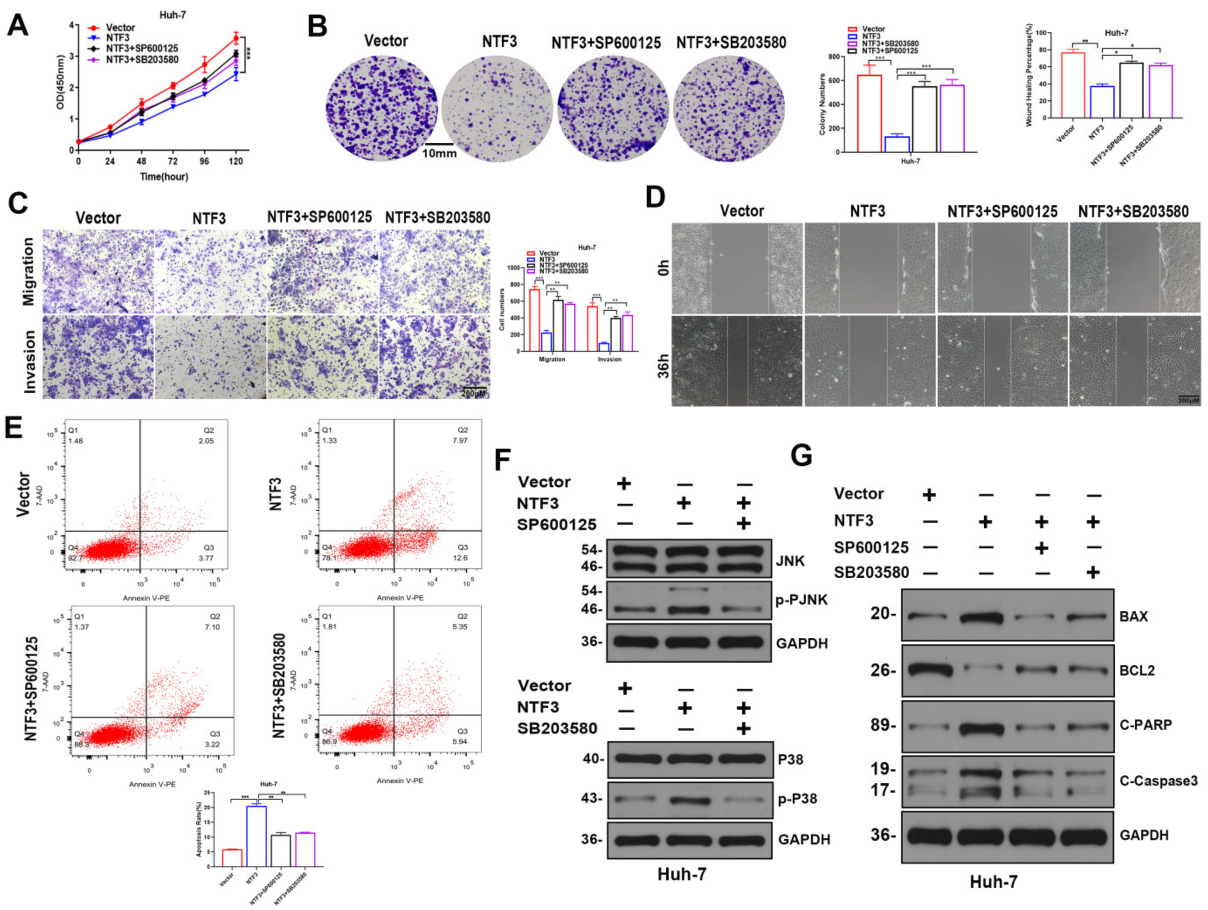

Fig. S4 JNK inhibitor SP600125 and P38 inhibitor SB20358 reversed the anti-tumor effect induced by NTF3. Huh-7 was categorized into four groups: 1) Vector, 2) NTF3, 3) NTF3+SP600125, and 4) NTF3+SB20358. After the cells were starved for 12 h, when the cell density reached about 40%, the high-glucose DMEM medium was replaced with 1% FBS, to which the JNK pathway inhibitor SP600125 (20 μM) and the P38 MAPK pathway inhibitor SB20358 (40 μM) were added for 2 days. **A, B** CCK8 and colony formation experiments suggested that the addition of inhibitors reversed the inhibitory effect of NTF3-induced proliferation. **C, D** Wound-healing, invasion, and migration experiments suggested that the addition of inhibitors reversed the invasion and migration effects of NTF3. **E** Set 4 groups in Huh-7 1) Vector, 2) NTF3 group, 3) NTF3+SP600125, 4) NTF3+SB20358; the changes of apoptosis were detected by flow cytometry. **F** 1) Vector, 2) NTF3 group, 3) NTF3+SP600125, 4) NTF3+SB20358; WB detects the changes of total and phosphorylated JNK and P38 levels. **G** 1) Vector 2) NTF3 group 3) NTF3+SP600125 4) NTF3+SB20358, WB detects the changes of apoptosis-related proteins in the four groups. **P* < 0.05; ***P* < 0.01; ****P* < 0.001.

Supplementary Table S1. Sequences of siRNA and shRNA in this study

Names	sequences
siRNA NC forward	5’-UUCUCCGAACGUGUCACGUTT-3’
siNTF3 NC reverse	5’-ACGUGACACGUUCGGAGA-3’
siNTF3 #1 forward	5’-GCAUCCAAGGUAACAACAUTT-3’
siNTF3 #1 reverse	5’-AUGUUGUUACCUUGGAUGCTT-3’
siNTF3 #2 forward	5’-CCAGAAGACUCGCUCAAUUTT-3’
siNTF3 #2 reverse	5’-AAUUGAGCGAGUCUUCUGGTT-3’
siNTF3 #3 forward	5’-CCCUCAUUAUUAAGCUGAUTT-3’
siNTF3 #3 reverse	5’-AUCAGCUUAAUAAUGAGGGTT-3’
siHDAC1#1 forward	5’-CCCAGAGGAGAAGAAAGAATT-3’
siHDAC1#1 reverse	5’-UUCUUUCUUCUCCUCUGGGTT-3’
siHDAC1#2 forward	5’-GGAGAAGCCAGAAGCCAAATT-3’
siHDAC1#2 reverse	5’-UUUGGCUUCUGGCUUCUCCTT-3’
siHDAC1#3 forward	5’-GAGGAAGAGUUCUCCGAUUTT-3’
siHDAC1#3 reverse	5’-AAUCGGAGAACUCUUCCUCTT-3’
siHDAC3#1 forward	5’-GCAUCUCUGCAAGGAGCAATT-3’
siHDAC3#1 reverse	5’-UUGCUCCUUGCAGAGAUGCTT-3’
siHDAC3#2 forward	5’-GCCGGUUAUCAACCAGGUATT-3’
siHDAC3#2 reverse	5’-UACCUGGUUGAUAACCGGCTT-3’
siHDAC3#3 forward	5’-GCCGCUACUACUGUCUGAATT-3’

siHDAC3#3 reverse	5’-UUCAGACAGUAGUAGCGGCTT-3’
sip75NTR#1 forward	5’-CCAACCAGACCGUGUGUGATT-3’
sip75NTR#1 reverse	5’-UCACACACGGUCUGGUUGGTT-3’
sip75NTR#2 forward	5’-GGAACAGCUGCAAGCAGAATT-3’
sip75NTR#2 reverse	5’-UUCUGCUUGCAGCUGUUCCTT-3’
sip75NTR#3 forward	5’-CCGAGCACAUAGACUCCUUTT-3’
sip75NTR#3 reverse	5’-AAGGAGUCUAUGUGCUCGGTT-3’
shNTF3#NC	TTCTCCGAACGTGTCACGT
shNTF3#1	CACTGACTTCAGAGAACAATA
shNTF3#2	CTCTCCCGTCAAACAATATTT
shNTF3#3	CGCTCAATTCCCTCATTATTA

Supplementary Table S2. Sequences of primers used for PCR in this study

Primer names	sequences
ACTB forward	5'-CATGTACGTTGCTATCCAGGC-3'
ACTB reverse	5'-CTCCTTAATGTCACGCACGAT-3'
NTF3 forward	5'-CCCCGCCCTTGTATCTCAT-3'
NTF3 reverse	5'-GACCTGGTGTCCCCGAAT-3'
p75NTR forward	5'-TGGCTGCTGTGGTTGTGG-3'
p75NTR reverse	5'-GAGGCTGTCTGCGTGTGG-3'
NTRK1 forward	5'-TCAAGGCACTGAAGGAGGC-3'
NTRK1 reverse	5'-CATGGGATCGGAGGAAGC-3'
NTRK2 forward	5'-CAAGAGGCTAAATCCAGTCCA-3'
NTRK2 reverse	5'-ACCAGGTTACCAACATCCCAA-3'
NTRK3 forward	5'-CTTTTGCCTGTGTCCTGTTGG-3'
NTRK3 reverse	5'-GGTGATGCCGTGGTTGATGT-3'
HDAC1 forward	5'-TCTGTTACTACTACGACGGGGAT-3'
HDAC1 reverse	5'-GCTTTGTGAGGGCGATAGATTTC-3'
HDAC3 forward	5'-TTACAAGCACCTTTTCCAGCC-3'
HDAC3 reverse	5'-GACATATTCAACGCATTCCCC-3'
NTF3 forward-ChIP	5'-ACGGCTTGCTTATTAGACA-3'
NTF3 reverse-ChIP	5'-CGCTCCTCACATCATCTC-3'

Supplementary Table S3. Primary antibodies used in this study

Antigens	Manufacturer	Catalog Number	Application
NTF3	Novus	NBP1-47892	1:5000 for WB
NTF3	Novus	NBP1-47892	1:100 for IHC
GAPDH	Proteintech	60004-1-Ig	1:20000 for WB
E-cadherin	Abcam	ab231303	1:500 for IHC
N-cadherin	Abcam	ab76057	1:500 for IHC
Bax	Cell signaling technology	#2772	1:2000 for WB
BCL-2	Abcam	ab196495	1:2000 for WB
Cleaved-Caspase3	Cell signaling technology	#9661	1:1000 for WB
Cleaved-PARP	Cell signaling technology	#5625	1:1000 for WB
P75NTR	Cell signaling technology	#8238	1:1000 for WB
JNK1/2	Cell signaling technology	#9252	1:1000 for WB
p-JNK1/2	Cell signaling technology	#4668	1:1000 for WB
P38	Cell signaling	#8690	1:1000 for WB

	technology		
p-P38	Cell signaling technology	#4511	1:1000 for WB
HDAC1	Abcam	ab280198	1:1000 for WB
HDAC3	Cell signaling technology	#85057	1:1000 for WB

TableS4 Relationship between NTF3 expression and clinicopathologic parameters of HCC patients

Characteristics	Number of cases	NTF3		P value
		High	Low	
**Age**				
<65	43	26	17	0.065
>=65	31	12	19	
**Gender**				
Female	30	14	16	0.443
Male	44	24	20	
**Tumor Size(cm)**				
<5	39	25	14	**0.021***
≥5	35	13	22	
**HBV infection**				
No	33	20	13	0.153
Yes	41	18	23	
**Tumor number**				
Single	45	26	19	0.168
Multiple	29	12	17	
**AFP(μg/L)**				
<400	43	26	17	0.623
≥400	31	12	19	
**Cirrhosis**				
No	31	16	15	0.97
Yes	43	22	21	
**TNM stage**				
I+II	56	36	20	**0.001*****
III+IV	18	2	16	
**BCLC stage**				
Low	51	36	15	**0.002****
High	23	2	21	
**Lymph metastasis**				
No	65	34	31	0.196
Yes	9	4	5	

BCLC: Barcelona Clinic Liver Cancer. *p < 0.05; **p < 0.01; ***p < 0.001.
The p values with significance are marked in bold.

Supplementary Table S5. Drugs used in this study

Name	Manufacturer	Catalog Number
3-Deazaneplanocin A hydrochloride	MedChemExpress	HY-12186
Decitabine	MedChemExpress	HY-A0004
Entinostat	MedChemExpress	HY-12163
RGFP966	MedChemExpress	HY-13909
Romidepsin	MedChemExpress	HY-15149
SB203580	MedChemExpress	HY-10256
SP600125	Selleck	S1460
